# Revisiting the musical reminiscence bump: insights from neurocognitive and social brain development in adolescence

**DOI:** 10.3389/fpsyg.2024.1472767

**Published:** 2024-10-01

**Authors:** Rishitha Kudaravalli, Nicholas Kathios, Psyche Loui, Juliet Y. Davidow

**Affiliations:** ^1^Department of Psychology, Northeastern University, Boston, MA, United States; ^2^Department of Music, Northeastern University, Boston, MA, United States

**Keywords:** music, social, reward, adolescence, development, emotion

## Abstract

Music listening is enjoyed across the lifespan and around the world. This has spurred many theories on the evolutionary purpose of music. The Music for Social Bonding hypothesis posits that the human capacity to make music evolved for the purpose of creating and preserving relationships between one another. Considering different time periods of music use across the lifespan, adolescence is especially a period of social reorientation away from family towards peers, characterized by new social bonds and increased prosocial behavior. This shift is accompanied by notable structural and functional changes in brain networks supporting reward processing and prosocial behavior. Reviewing the extant literature on developmental cognitive neuroscience and adolescent music use, we propose that neurocognitive changes in the reward system make adolescence an ideal developmental time window for investigating interactions between prosocial behavior and reward processing, as adolescence constitutes a time of relative increase in music reward valuation. Testing this hypothesis may clarify our understanding of developmental trajectories in music reward valuation, and offer insights into why music from adults’ adolescence holds a great deal of personal significance.

## Introduction

Despite no obvious evolutionary advantage, music is enjoyed by listeners across the lifespan and around the globe (Dubé and Le Bel, 2003). Adult listeners show increased preference for and personal memories associated with music from their adolescence and young adulthood (the “reminiscence bump” effect; Krumhansl and Zupnick, 2013; Krumhansl, 2017; Jakubowski et al., 2020). Why we enjoy listening to music (Loui et al., 2017; Mas-Herrero et al., 2013; Salimpoor et al., 2013) and show this reminiscence bump effect (Lamont and Loveday, 2020; Peck and Grealey, 2020) has been the topic of much research. Neuroscientific studies have generally converged on the idea that music listening involves interactions between the reward system, including the medial prefrontal cortex and striatum (similar to primary rewards such as food; Mas-Herrero et al., 2021), and the with the auditory cortex (Quinci et al., 2022; Salimpoor et al., 2013). The Music for Social Bonding Hypothesis argues that human capacity to make music evolved for the purpose of creating and preserving relationships between one another (Savage et al., 2020). In this view, the engagement of the reward system through the enjoyment of music (Quinci et al., 2022; Salimpoor et al., 2013) leads to positive emotional affiliations with those with whom music is shared.

Social bonds play an important role in human development across cultures and can even support healthy aging (Antonucci et al., 2019). Adolescence, which can be defined as the time encompassing the onset of puberty until independence is achieved (Casey et al., 2015), is a period of “social reorientation” away from caregivers (Faust et al., 2020) and towards peers (Cosme et al., 2022; Crone and Dahl, 2012; Larson, 2001; Nelson et al., 2005). This shift in the importance of peer relationships intensifies adolescents’ social sensitivity, or the attention, salience and emotional response directed towards processing information related to social evaluations and social standing (Somerville, 2013). At the same time, adolescents report listening to music in social contexts more often than older age groups (Bonneville-Roussy et al., 2013) and even establish friendships according to musical preferences (Franken et al., 2017; Kistler et al., 2010; Miranda, 2013; North and Hargreaves, 1999; Selfhout et al., 2009; ter Bogt et al., 2017). Here, we review key neurocognitive developmental changes in the social brain, specifically in the reward system, during adolescence, and interpret them in light of the existing literature on adolescent music usage and consumption. Together, we argue that these suggest the existence of a developmental increase in music reward valuation during adolescence, which can provide unique insight into the relationship between music and memory across the lifespan.

## Neurocognitive development of social evaluation engages reward processing in adolescence

The reorientation of adolescents’ social world is accompanied by ongoing changes in social cognition. Fundamental skills which support mentalizing, or understanding the mental states of others, are apparent as early as two years old (for a review, see Carlson et al., 2013). Popularly employed methods used to study mentalizing typically show ceiling-level performance at around five years old (Wellman et al., 2001), suggesting that this ability is readily developed by the end of childhood. However, more complex mentalizing tasks which require individuals to take others’ perspectives while inhibiting their own (i.e., perspective-taking) indicate otherwise. These studies show that adolescents make more errors compared to adults (Dumontheil et al., 2010; Symeonidou et al., 2016), with older adolescents (17 year-olds) outperforming younger adolescents (12- and 15-year-olds; Humphrey and Dumontheil, 2016). These findings suggest that higher-level mentalizing skills improve throughout adolescence until reaching adultlike abilities.

Studies of brain structure and function during adolescence complement this interpretation. Neuroimaging studies have identified a network of regions that support mentalizing (known as the “social brain”), which includes the superior temporal sulcus, temporoparietal junction, and medial prefrontal cortex (Amodio and Frith, 2006; Lieberman et al., 2019; Rilling and Sanfey, 2011; Saxe et al., 2009). This network is active in youth while listening to descriptions of others’ mental states (Gweon et al., 2012; Mukerji et al., 2019; Saxe et al., 2009), watching cartoons (Brunet et al., 2000; Gallagher et al., 2000; Sebastian et al., 2012), and even in the mere presence of peers (van Hoorn et al., 2016). These regions undergo notable structural changes throughout adolescence, with decreases in cortical thickness and gray matter volume in the superior temporal sulcus, temporoparietal junction, and dorsal medial prefrontal cortex (Mills et al., 2014). Adolescents also show increased activation of the medial prefrontal cortex compared to adults during mentalizing tasks (Blakemore et al., 2007; Gunther Moor et al., 2012; Sebastian et al., 2012), which some interpret as adolescents employing different mentalizing strategies compared to adults (Blakemore, 2008).

Such developments in social cognition and the social brain come at a time of increased prosocial behavior, or actions which serve the goal of helping others. Numerous longitudinal studies show a relative increase in such behavior throughout adolescence (Crocetti et al., 2016; te Brinke et al., 2023; Van der Graaff et al., 2018). Importantly, the development of mentalizing abilities is linked to adolescent prosocial behavior (Blankenstein et al., 2020; Hall et al., 2021). For instance, perspective taking performance in adolescence is positively correlated with self-reported prosocial behavior (Tamnes et al., 2018), more giving behavior (van de Groep et al., 2020), and higher levels of trust and reciprocity in group cooperation tasks (Fett et al., 2014). As a result, adolescents’ increased engagement in prosocial behavior may be due, in part, by their improved ability to understand the wants and needs of others.

Engaging in prosocial behavior elicits activation of regions within the reward system, which consists of the ventral tegmental area, ventral striatum, and ventral medial prefrontal cortex (Decety et al., 2004; Rilling and Sanfey, 2011; Telzer, 2016; Telzer et al., 2010; van de Groep et al., 2020; Westhoff et al., 2021; Zaki and Mitchell, 2013). This network—and ventral striatum in particular—plays an important role in the anticipation of and reactivity to a wide array of rewarding experiences (Pagnoni et al., 2002; Schultz et al., 1997), including monetary (Galvan et al., 2006; Van Leijenhorst et al., 2010) and social rewards (such as smiling faces; Somerville et al., 2011, and peer evaluation; Cheng et al., 2020; Guyer et al., 2009; Jones et al., 2014; Sherman et al., 2016). This system also shows protracted development into late adolescence (Dahl, 2004; Padmanabhan and Luna, 2014; Spear, 2000; Wahlstrom et al., 2010). Compared to children and adults, adolescents show heightened activity of the ventral striatum, a region longitudinally linked to greater self-reports of reactivity to rewards (Urošević et al., 2012), during the receipt of a reward compared to children and adults (Braams et al., 2015; Cohen et al., 2010; Ernst et al., 2005; Galvan et al., 2006; Schreuders et al., 2018; Silverman et al., 2015; Van Leijenhorst et al., 2010). This has led some to hypothesize that adolescents spend more time with peers due to a relative increase in the value of social interactions during this time (Foulkes and Blakemore, 2016). Indeed, adolescents show greater activation in the ventral striatum compared to children (6–12 year olds) and adults (18–29) in response to positive (happy facial expressions) compared to neutral (calm facial expressions) social cues (Somerville et al., 2011).

Some evidence also suggests that the value of non-social rewards increase in the presence of peers during adolescence (Chein et al., 2011; Powers et al., 2022; Weigard et al., 2014). For instance, the ventral striatum and temporoparietal junction show greater activation while adolescents make decisions while being told they were being observed by peers compared to parents (van Hoorn et al., 2018). The ventral striatum also responds to rewards received by others (Braams and Crone, 2017), and in adults this activity scales with how close individuals feel with the recipient (Hackel et al., 2017; Morelli et al., 2015). The magnitude of this vicarious response is positively correlated with everyday prosocial behavior (Morelli et al., 2018). The engagement of the ventral striatum during prosocial behavior may thus motivate adolescents to partake in such behavior (Crone et al., 2022). This interpretation is consistent with results that show adolescents typically act prosocially towards their friends (Berndt and Hoyle, 1985; Güroğlu et al., 2014; Padilla-Walker et al., 2018). In sum, improvements in mentalizing and relative increases in reactivity to rewarding experience make adolescence a uniquely suited time window for investigating interactions between prosocial behavior and reward processing.

## Music reward valuation

Advances in understanding the brain structures underlying pleasurable music listening experiences show that music engages the reward system similar to other primary rewards (Mas-Herrero et al., 2021). Broadly speaking, enjoyable music listening experiences typically involve interactions between these structures (including the ventral striatum) and auditory and frontal cortices (Mas-Herrero et al., 2021; Salimpoor et al., 2011; Salimpoor et al., 2013; Belden et al., 2023). Activity of this network scales with preference listeners have with music (Salimpoor et al., 2013), and evidence for the causality of this network transcranial magnetic stimulation studies (Mas-Herrero et al., 2018). As a result, how music acquires reward value has been the focus of much research. One line of work has specifically investigated how music acquires intrinsic reward value (i.e., the enjoyment of music in and of itself). The Predictive Coding of Music Model (Vuust et al., 2022) argues that listening to music involves an ongoing process of generating predictions about upcoming musical elements, with the goal of reducing mismatches between listeners’ predictions and experience of the music (i.e., prediction errors). Rewarding feelings that accompany the experience of musical prediction errors thus serve as the basis for the updating of listeners’ musical predictions.

To date, there have been few cross-sectional age comparisons of the experience of music-related reward. Belden et al. (2023) demonstrated age-related differences in functional connectivity during music listening. In this study, both younger (18–23 years old) and older adults (54–89 years old) completed a music listening fMRI task during which they listened to self- and researcher-selected music. During this task, the younger adult cohort exhibited greater functional connectivity between the medial prefrontal cortex and auditory cortex compared to the older adults (Belden et al., 2023). These results mirror age-related declines in self-reported music enjoyment across adulthood (Belfi et al., 2022). Only two studies, to our knowledge, have investigated functional differences in brain activity in populations younger than young adults (Berns et al., 2010; Fasano et al., 2022). Berns et al. (2010) exposed adolescent participants (ages 12–17 years) to 15 s music clips, after which they rated each on liking and familiarity. Activity in the striatum was correlated with participants’ liking ratings. Similarly, Fasano et al. (2022) identified a network involving the medial orbitofrontal cortex and ventromedial prefrontal cortex, which reliably subserved music listening in preadolescents (ages 10–11 years). Importantly, these studies show that music listening engages frontostriatal circuitry similar to adults. While comparisons across ages in this experience remain to be tested, these studies suggest that musical reward may also elicit a relative increase in the engagement of this circuitry during adolescence.

Responses to the Barcelona Music Reward Questionnaire (Mas-Herrero et al., 2013), a self-report measure of individual differences in sensitivity to musical reward, show that sharing music with others is another source musical enjoyment across cultures (the “Social Reward” dimension; Mannino et al., 2024; Wang et al., 2023). While Belfi and Loui (2020) found that scores on this measure remain constant across adulthood, adolescents’ sensitivity to social information (Guyer et al., 2009; Somerville, 2013) offers a theoretical basis as to why this construct may show age-related differences earlier in development. Indeed, musical training between the ages of 10–14 years old is linked to greater sensitivity to this source of musical pleasure (Lippolis et al., 2023). Berns et al. (2010) allowed participants to update their liking ratings after being presented with “popularity scores” of each music clip. Participants were likely to update their liking ratings in accordance with these popularity metrics (e.g., popular songs were liked more; Berns et al., 2010), providing evidence of peer influence on adolescents’ subjective music enjoyment, even when they are not physically present. A recent study has also demonstrated that merely telling adult participants that others were listening to the same music at the same time boosted the subjective pleasure participants felt listening to that music (Curzel et al., 2024). Together, these studies suggest that sharing music with others may be a salient source of musical reward for adolescents and that doing so may increase overall pleasure during music listening.

## Extant work on adolescent music usage

Reports of adolescent music usage are broadly consistent with the idea that listeners are particularly responsive to both the implicit and social rewards of music during this time. Cross-sectional studies show the importance attributed to music is higher in adolescent listeners (ages 13–18 years old) compared to older age groups (Bonneville-Roussy et al., 2013; Lamont and Hargreaves, 2019). Further, music listening nearly doubles for 11–14 year-olds listeners compared to those 8–10 years old, and this engagement continues to increase as listeners reach 18 years of age (Roberts and Henriksen, 2004). On the other hand, a large-scale study on music making in teenagers in the UK and Germany showed that about 50% of all students drop out of music lessons and other musical activities by the time they turn 17 years old, with most students quitting between the ages of 15 and 17 (Ruth and Müllensiefen, 2021). Thus, music listening appears to increase during adolescence, even while active music making decreases. This may be indicative of a general increase in media consumption during this time window; however, adolescents prioritize music listening over other forms of entertainment, such as reading a book, going shopping, or playing computer games (North et al., 2000). This suggests that adolescents’ affinity for music is likely due to affordances of music listening not offered by other forms of media.

A unique feature of music is its ability to create interpersonal synchrony, or time-locked coordinated movement (Trainor and Cirelli, 2015). Such synchrony to music signals group membership (Tarr et al., 2014; Hove and Risen, 2009) and motivates prosocial behavior (Wiltermuth and Heath, 2009; Stupacher et al., 2017; Cirelli et al., 2014), highlighting the social nature of music. At the same time, adolescents report listening to music in more social contexts compared to older individuals (Bonneville-Roussy et al., 2013) and form peer groups and close affiliations around shared musical preferences (Bakagiannis and Tarrant, 2006; Franken et al., 2017; Miranda and Claes, 2009; Selfhout et al., 2009). Further, adolescents attribute the ability to use music to curate their identities, which likely serve as the basis for relationships created through shared musical preferences, as one reason why music holds such great importance during this time (North et al., 2000). Such results suggest that increases in social sensitivity during adolescence may account for the importance placed on music during this time. Consistent with studies that directly manipulate interpersonal synchrony (Wiltermuth and Heath, 2009; Stupacher et al., 2017; Cirelli et al., 2014), affiliations based solely on musical preferences elicit prosocial behavior in adolescents (Bakagiannis and Tarrant, 2006; North et al., 2004).

Adolescent music usage, paired with ongoing neurocognitive development of brain systems for reward and social processes, make this an insightful window for understanding the role of rewarding music listening experiences in the Music for Social Bonding hypothesis. We propose that heightened reactivity to both intrinsic and social rewards of music listening support adolescent prosocial behavior towards those they share music with. Specifically, adolescents may initially be motivated to listen to music with others due to the boost that social music listening offers to musical pleasure (Curzel et al., 2024), and adolescents’ increased reactivity to rewarding stimuli (Galvan et al., 2006; Van Leijenhorst et al., 2010; Galván and McGlennen, 2013) especially among peers (Chein et al., 2011; Weigard et al., 2014). According to the Music for Social Bonding Hypothesis (Savage et al., 2020), such boosts in musical pleasure shared with individuals may, in return, contribute to strong social bonds built around music during adolescence. The shift towards social music listening during adolescence may thus be supported by the ongoing development of reward processing during this time period. These affiliations established through music may then be the basis for the experience of vicarious rewards which motivate prosocial behavior. Similar mechanisms likely support prosocial behavior elicited between individuals with shared musical preferences (Bakagiannis and Tarrant, 2006; North et al., 2004). The social inferences that individuals may make about each other (e.g., what kind of clothes someone might wear or activities they might enjoy), based solely upon others’ musical preferences, may be used as information to cue individuals to form new relationships (North and Hargreaves, 1999; Rentfrow and Gosling, 2006).

## Discussion: evaluating accounts of the reminiscence bump effect

A core tenet of our proposal is that adolescence may be a time during which individuals show a socially-boosted increase in the rewards of music listening. While this offers implications for how we might understand music and prosocial behavior, it may also shed light on other music-related phenomena. One such instance is the “reminiscence bump” effect, which is a robust finding in autobiographical memory, or recollections of one’s own experiences (Koppel and Berntsen, 2015; Rubin et al., 1986). This describes adults’ general tendency to recall the most autobiographical memories from when they were between the ages of 10–30 years old (Koppel and Berntsen, 2015; Rubin et al., 1986). This effect has been illustrated in response to a number of cues, such as words (Crovitz and Schiffman, 1974; Rubin and Schulkind, 1997), pictures (Willander and Larsson, 2006), and music (Jakubowski et al., 2020; Krumhansl, 2017). Studies have shown that the timing of this reminiscence bump effect differs between these cues, suggesting contributions from different mechanisms supporting this effect across modalities (Koppel and Berntsen, 2015).

In the realm of music, the reminiscence bump effect specifically describes a relative increase in the amount of personally significant memories associated with or the amount of spontaneous autobiographical memories evoked by music from listeners’ adolescence and early adulthood (Jakubowski et al., 2020; Krumhansl, 2017; Krumhansl and Zupnick, 2013; Platz et al., 2015). Studies which illustrate the reminiscence bump in music typically use popular music cues and calculate “song-specific age” of each cue (Jakubowski et al., 2020; Krumhansl, 2017; year of music popularity minus participants’ birth year) as a proxy for developmental exposure. For example, Jakubowski et al. (2020) found that participants across adulthood (ages 18–82 years old) reported having the most memories associated with music with a song-specific age between 10–19 years (with a peak around 14 years). A notable departure of this approach compared to other methods which characterize this effect is that it emphasizes the timing of listeners’ developmental exposure to popular music cues and not necessarily the timing of the evoked memories. Though this presents a confound in comparing the music-related bump to the general reminiscence bump, it points to developmental processes within the musical reminiscence bump time period giving rise to this effect. However, using song-specific age as a proxy for developmental exposure to music can be limited by the fact that listeners may not be first exposed to popular music at the time it was popularized. Here, we argue that investigations of music reward valuation across adolescence may provide additional evidence to link ongoing developmental processes to the musical reminiscence bump effect with higher specificity and experimental control.

Two theoretical accounts which emphasize ongoing developmental processes during the reminiscence bump time period exist to explain the origin of this effect (for a review of these accounts, see Munawar et al., 2018). Some have theorized that the ongoing importance of self and group identity construction during the reminiscence bump window is the origin of the reminiscence bump effect (i.e., the “Identity Formation” account; Conway, 2005; Munawar et al., 2018; Peck and Grealey, 2020). This account argues that experiences acquired during this period are integrated into an individual’s lifelong narratives and are thus often recalled later in life as an understanding or explanation of one’s identity (Conway, 2005). As a result, memories from the reminiscence bump time period remain durable and easily recalled across the lifespan. Given the emphasis some adolescents place on music listening for their identity (Franken et al., 2017; Kistler et al., 2010; Miranda and Claes, 2009; North and Hargreaves, 1999; Selfhout et al., 2009; ter Bogt et al., 2017), this account fits nicely with why individuals may experience a reminiscence bump in response to music. A similar explanation for this effect which emphasizes ongoing developmental processes is the Cognitive Abilities or Enhanced Encoding account (Janssen and Murre, 2008; Janssen et al., 2007). This account argues that individuals have an increased ability to encode memories during this time due to improvements in cognitive and neural functions which may cause the bump.

Other accounts of the reminiscence bump effect emphasize the nature of the experiences during the reminiscence bump time period and subsequent effects on memory retrieval. The Cultural Lifescript account, for instance, argues that the reminiscence bump time window is made up of stereotypical episodes consisting of multiple culturally significant events in a specific order (e.g., start high school, go to prom, graduate, go to college, etc.). While these lifescripts may vary cross-culturally, individuals’ awareness of this timeline results in events occurring within the reminiscence bump time period as both personally and culturally significant (Berntsen and Rubin, 2002). As a result, these memories are both strongly encoded and subsequently recalled often throughout the lifespan, resulting in their lifelong durability. Similarly, the Cognitive account states that increased exposure to novel experiences during this time period, such as one’s first date or first time driving, leads to increased lifelong memory retention of these events. Because these are first-time experiences, these memories serve as reference points for similar events later in life (Pillemer, 2001), and are thus not only retained because they are originally novel experiences, but also because individuals continue to recall them as they get older. Together, these four accounts are not mutually exclusive, but they do place different emphases on memory encoding and retrieval which are all likely at play in producing the musical reminiscence bump. Given methods used to elicit this effect emphasize likely developmental exposure to music cues, we focus our argument on the links between the proposed increase in music reward valuation during adolescence and accounts which emphasize ongoing developmental processes, i.e., the Cognitive Abilities account.

A consistent finding within the music-related reminiscence bump literature is that this effect is often associated with reports of stronger emotional responses (Schulkind et al., 1999; Platz et al., 2015) and greater preference for music from this time window (Holbrook and Schindler, 1989; Janssen et al., 2007; Kathios et al., 2023; Krumhansl, 2017). For instance, Janssen et al. (2007) instructed participants to report their favorite movies, books, and music records, as well as when they first encountered and the last time they had seen, read, or listened to each. Of these mediums, the amount of participants’ favorite records encountered during the reminiscence bump (ages 11–25) showed the biggest peak. This common finding has led some to hypothesize that increased preference for and emotional reactions to music from the reminiscence bump time period is due to the developmentally-specific associations listeners have for this music (Lamont and Loveday, 2020). However, heightened responsivity to the rewards of music listening *during* the reminiscence bump time period may also account for this lifelong preference. In fact, such increased responses to musical reward during this time period might be causally involved in the reminiscence bump effect.

Rewarding experiences generally enhance memory of associated information (Bialleck et al., 2011; Mather and Schoeke, 2011), perhaps more so in adolescence (Davidow et al., 2016; Nussenbaum and Hartley, 2021; Rosenbaum et al., 2022). Consistent with the Cognitive Abilities or Enhanced Encoding accounts of the reminiscence bump effect (Janssen and Murre, 2008; Janssen et al., 2007), heightened reactivity to musical reward may optimize the effect of musical reward on memory during this time. Only a handful of studies have investigated the relationship between reward and memory in music (Cardona et al., 2020; Curzel et al., 2024; Ferreri et al., 2021; Ferreri and Rodriguez-Fornells, 2017). These studies show that pleasure experienced during music listening is positively associated with memory outcomes, such as remembering the music itself (Ferreri et al., 2021; Ferreri and Rodriguez-Fornells, 2017; Curzel et al., 2024) and recalling words encoded during music listening (Cardona et al., 2020). Interestingly, in three of these studies (Ferreri and Rodriguez-Fornells, 2017; Ferreri et al., 2021; Cardona et al., 2020), this effect was strongest for those who reported high sensitivity to musical reward. These individual differences results are consistent with our proposition that developmental changes in music reward valuation *within* an individual (specifically during adolescence) may result in improved associated memory encoding during this time window. Such findings highlight the relevance of our proposal that adolescents may be a period of heightened music reward valuation to memory.

Conversely, our proposal may imply that adolescents who are uninterested in music may not show a musical reminiscence bump effect later in life. However, it is important to note that music-evoked autobiographical memories tend to be social in nature (Jakubowski and Ghosh, 2021; Janata et al., 2007). This, paired with the increase in social music listening in adolescence (Bonneville-Roussy et al., 2013), suggests that these individuals may still show a bump as adults due to incidental music exposure in social contexts. Though adolescents report a notable degree of dropout of music lessons (Ruth and Müllensiefen, 2021), it may be the case that music listening (but not recurrent music lessons) elicits specific episodic music-evoked autobiographical memories which develop alongside the development of theory of mind (Kristen-Antonow, 2019) and reward processing in adolescence.

## Conclusion

Adolescence is a time of intense changes, both in the social environment and in the brain. It stands to reason that music use during this time period may undergo developmentally significant changes as well. The engagement of neural systems for processing rewards during music listening is hypothesized to play a critical role relating enjoyable music listening experiences to prosocial outcomes (Savage et al., 2020). Extant studies suggest that the importance of music in adolescence is driven by a combination of intrinsic enjoyment and extrinsic social motivations. We propose that developmental changes in the reward system and the social brain may support heightened brain and behavioral responsivity to musical reward during adolescence. Specifically, we argue that greater sensitivity to the intrinsic and social rewards of music listening may motivate adolescents to share music with others, thus facilitating greater social bonds and prosocial behavior between those who share music with one another. Finally, we highlight how investigating this hypothesis may also provide insight into why music from listeners’ adolescence is preferred and elicits more autobiographical memories compared to music from outside this time period. Though current research on the developmental trajectory of music reward valuation across adolescence is limited, inquiries in this area hold relevance beyond reward processing to both prosocial behavior and lifelong memories.

## Data Availability

The original contributions presented in the study are included in the article/supplementary material, further inquiries can be directed to the corresponding author.
